# Human lymphocytes mobilized with exercise have an anti-tumor transcriptomic profile and exert enhanced graft-versus-leukemia effects in xenogeneic mice

**DOI:** 10.3389/fimmu.2023.1067369

**Published:** 2023-04-03

**Authors:** Helena Batatinha, Douglass M. Diak, Grace M. Niemiro, Forrest L. Baker, Kyle A. Smith, Tiffany M. Zúñiga, Preteesh L. Mylabathula, Michael D. Seckeler, Branden Lau, Emily C. LaVoy, Michael P. Gustafson, Emmanuel Katsanis, Richard J. Simpson

**Affiliations:** ^1^ School of Nutritional Sciences and Wellness, The University of Arizona, Tucson, AZ, United States; ^2^ Department of Pediatrics, The University of Arizona, Tucson, AZ, United States; ^3^ Cancer Center, The University of Arizona, Tucson, AZ, United States; ^4^ University of Arizona Genetics Core, The University of Arizona, Tucson, AZ, United States; ^5^ Department of Health and Human Performance, University of Houston, Houston, TX, United States; ^6^ Department of Laboratory Medicine and Pathology, Mayo Clinic in Arizona, Phoenix, AZ, United States; ^7^ Department of Immunobiology, The University of Arizona, Tucson, AZ, United States

**Keywords:** exercise immunology, NSG mice, single cell transcriptomics, donor lymphocyte infusions, adoptive cell therapy, cancer, immunotherapy, graft-versus-host disease

## Abstract

**Background:**

Every bout of exercise mobilizes and redistributes large numbers of effector lymphocytes with a cytotoxic and tissue migration phenotype. The frequent redistribution of these cells is purported to increase immune surveillance and play a mechanistic role in reducing cancer risk and slowing tumor progression in physically active cancer survivors. Our aim was to provide the first detailed single cell transcriptomic analysis of exercise-mobilized lymphocytes and test their effectiveness as a donor lymphocyte infusion (DLI) in xenogeneic mice engrafted with human leukemia.

**Methods:**

Peripheral blood mononuclear cells (PBMCs) were collected from healthy volunteers at rest and at the end of an acute bout of cycling exercise. Flow cytometry and single-cell RNA sequencing was performed to identify phenotypic and transcriptomic differences between resting and exercise-mobilized cells using a targeted gene expression panel curated for human immunology. PBMCs were injected into the tail vein of xenogeneic NSG-IL-15 mice and subsequently challenged with a luciferase tagged chronic myelogenous leukemia cell line (K562). Tumor growth (bioluminescence) and xenogeneic graft-versus-host disease (GvHD) were monitored bi-weekly for 40-days.

**Results:**

Exercise preferentially mobilized NK-cell, CD8+ T-cell and monocyte subtypes with a differentiated and effector phenotype, without significantly mobilizing CD4+ regulatory T-cells. Mobilized effector lymphocytes, particularly effector-memory CD8+ T-cells and NK-cells, displayed differentially expressed genes and enriched gene sets associated with anti-tumor activity, including cytotoxicity, migration/chemotaxis, antigen binding, cytokine responsiveness and alloreactivity (e.g. graft-versus-host/leukemia). Mice receiving exercise-mobilized PBMCs had lower tumor burden and higher overall survival (4.14E+08 photons/s and 47%, respectively) at day 40 compared to mice receiving resting PBMCs (12.1E+08 photons/s and 22%, respectively) from the same donors (p<0.05). Human immune cell engraftment was similar for resting and exercise-mobilized DLI. However, when compared to non-tumor bearing mice, K562 increased the expansion of NK-cell and CD3+/CD4-/CD8- T-cells in mice receiving exercise-mobilized but not resting lymphocytes, 1-2 weeks after DLI. No differences in GvHD or GvHD-free survival was observed between groups either with or without K562 challenge.

**Conclusion:**

Exercise in humans mobilizes effector lymphocytes with an anti-tumor transcriptomic profile and their use as DLI extends survival and enhances the graft-versus-leukemia (GvL) effect without exacerbating GvHD in human leukemia bearing xenogeneic mice. Exercise may serve as an effective and economical adjuvant to increase the GvL effects of allogeneic cell therapies without intensifying GvHD.

## 1 Introduction

Regular physical activity is widely regarded as contributing to lower cancer risk, improving outcomes in cancer survivors, and acting as an adjuvant for several types of cancer therapy (1–3). This assertion has come from a plethora of epidemiological studies linking physical activity to a reduction in cancer occurrence, randomized controlled trials demonstrating beneficial effects of exercise on treatment associated adverse effects, and preclinical studies showing increased tumor suppression in rodents exposed to exercise (4, 5).

Each bout of exercise evokes a catecholamine dependent mobilization and redistribution of effector lymphocytes (e.g. Natural Killer-cells, γδ T-cells, CD8+ T-cells) that has been purported to improve long-term immunosurveillance by increasing recognition and destruction of premalignant cells in the initial stages of tumor development, and contributing to the suppression of tumor growth (6, 7). Indeed, exercise has been shown to increase NK-cell and CD8+ T-cell tumor infiltration and suppress tumor growth in several murine cancer models (8, 9). These tumor infiltrating lymphocytes are similar in phenotype to those mobilized to blood with each exercise bout, indicating that exercise-mobilized lymphocytes could be harnessed for adoptive cell therapies (1,4). Donor lymphocyte infusions (DLI) are commonly delivered after allogeneic stem cell transplantation (allo-HSCT) to prevent and/or treat leukemic relapse. Unfortunately, the success rate of DLI to induce remission remains low (10) and the procedure also increases the risk of graft-versus-host disease (GvHD) – a potentially fatal condition whereby donor T-cells attack healthy tissues in the host causing significant morbidity (11). As such, using exercise mobilized DLI could have therapeutic ramifications if they are found to exert enhanced graft versus leukemia (GvL) effects without increased GvHD.

The characterization of exercise-mobilized lymphocytes has hitherto been limited to just a few surface markers or simple *in vitro* assays to determine function (e.g. cytotoxicity, proliferation), with the latter oftentimes confounded by cell composition shifts that have been evoked by exercise (3). No study, to our knowledge, has transcriptionally profiled exercise-mobilized lymphocytes in humans at single cell resolution or assessed their ability to control human leukemic growth in a whole organism.

The aim of this study, therefore, was to deeply interrogate the transcriptional and phenotypic profiles of exercise mobilized lymphocytes in humans and test their ability to reduce leukemic burden and xenogeneic GvHD in immunodeficient (NSG) mice. We hypothesized that (i) exercise would preferentially mobilize effector lymphocytes to the blood compartment with an enrichment of multiple transcriptional programs associated with anti-tumor activity; and (ii) that exercise mobilized lymphocytes would extend survival and reduce tumor burden without increasing GvHD after adoptive transfer to human leukemia bearing NSG mice. We report for the first time that exercise-mobilized peripheral blood mononuclear cells have differential gene expression (DGEs) and enriched gene sets associated with anti-tumor activity, particularly within effector memory subsets of CD8+ T-cells and NK-cells. Moreover, adoptively transferring these cells into human leukemia-bearing NSG mice prolonged survival by augmenting the GvL effect without exacerbating GvHD, underscoring the potential for exercise-mobilized lymphocytes to enhance the efficacy of allogeneic adoptive cell therapies.

## 2 Materials and methods

### 2.1 Participants

Sixteen healthy adults, (6 females, 10 males) participated in this study (Mean ± SD: age: 26.8 ± 5.2 yr; weight: 69.7 ± 13.6 kg; Height: 171.2 ± 15 cm; BMI: 23.2 ± 2 (kg/m^2^). Written informed consent and medical history were obtained from each volunteer after proper explanation of the procedures and risks. Only participants classified as ‘low risk’ in accordance with the risk stratification guidelines published by the American Heart Association and the American College of Sports Medicine (AHA/ACSM criteria) were enrolled (12). The study was approved by the Human Subjects Protection Program at the University of Arizona (#1801161041).

### 2.2 Exercise trials and blood sampling

All participants were asked to visit the laboratory on two separate occasions with one-two weeks separating each visit. Both laboratory visits occurred between 08:00 and 10:00 local time. Participants were instructed to arrive at the lab following an 8-12h overnight fast (water could be consumed during the fasting period). Additionally, participants were required to refrain from engaging in vigorous physical activity/exercise for 24 hours prior to each visit.

For Visit 1, subjects were asked to complete a maximal exercise protocol on an indoor stationary bicycle (Velotron, Quarq Technology, San Diego, CA). Heart rate, ECG activity and respiratory gas exchange was recoded continuously throughout the test (Cosmed CPET, Rome, Italy). Subjects began cycling at 50 Watts for females and 75 Watts for males, and the power was increased by 15 Watts every minute until volitional exhaustion. Ventilatory threshold, determined as the point corresponding to a rise in the ventilatory equivalent of VO_2_ (V_E_/VO_2_) without a concurrent rise in the ventilatory equivalent of VCO_2_ (V_E_/VCO_2_), and the determined VO_2max_ were used to assign workload for Visit 2.

For Visit 2, an intravenous catheter was inserted and blood was collected before exercise in vacutainer tubes treated with ethylene-diamine-tetra-acetic acid (EDTA) or acid citrate dextrose (ACD) (Becton-Dickinson, USA). Subjects were prepared for continuous heart rate, ECG and respiratory gas exchange collections then asked to cycle at either a power wattage corresponding to 15% above the ventilatory threshold for 30-minutes, or to perform 20-minutes of graded exercise, consisting of four incremental 5-minute stages at power outputs corresponding to 50%, 60%, 70%, and 80% of the individual VO_2max_. A final venous blood sample was collected during the last 3-5 minutes of the exercise protocol (during-exercise).

### 2.3 Blood processing and analysis

Complete blood counts were performed on blood collected in EDTA tubes using an automated hematology analyzer (Beckman Coulter, Indianapolis, IN). Whole blood samples from EDTA tubes were labeled with monoclonal antibodies to detect and enumerate leukocyte and progenitor cell subpopulations in blood by flow cytometry (MACSQuant 10, Miltenyi Biotec, San Diego, CA). All immune cell phenotypes were determined within the peripheral blood mononuclear cell (PBMC) gate based on forward and side scatter using FlowLogic (Inivai Technologies, Mentone Victoria, Australia). Single color tubes on whole blood samples were used for electronic color compensation and Fluorescence Minus One (FMOs) were used to set proper gating. A minimum of 50,000 PBMCs were collected for analysis. The flow cytometry antibody panels used are shown in **Supplementary Table 1**. Peripheral blood mononuclear cells (PBMCs) were isolated from blood collected in ACD tubes and cryopreserved in liquid nitrogen at a concentration of 10x10^6^ cells/mL freezing media (90% FBS, 10% DMSO) until further use in animal experiments.

### 2.4 RNAseq

Isolated PBMCs were resuspended in a PBS/RNAlater solution and delivered to the University of Arizona Genetics Core for single cell RNA sequencing (scRNAseq) analysis using the 10x Genomics platform. 5’ RNA whole transcriptome libraries were generated using the “10xGenomics Chromium Next GEM Single Cell 5’ reagents kit v2”, following recommended guidelines. The gene expression libraries were quantified, normalized, pooled, and sequenced on an Illumina NextSeq500 sequencer. FastQ files were converted into expression matrices using the “cellranger count” function provided by Cell Ranger (10x Genomics Cell Ranger 6.0.1) and unfiltered matrices were imported into R, version 4.1.0. Empty droplets were identified and removed using the emptyDrops function found in the DropletUtils package. Reads with a high percentage of mitochondrial content were identified and removed using the perCellQCMetrics function provided by scuttle. To analyze and visualize gene expression on a per cell basis, principal component analysis (PCA) and uniform manifold approximation and projection (UMAP) clustering was performed using Seurat, version 4.0.5. Differentially expressed genes were then detected using the FindMarkers function, with a log2 fold cutoff of 0, in Seurat. For each differential expression analysis comparison, gene set enrichment analysis (GSEA), with a false discovery rate of (0.25), was performed and annotated to both Kyoto Encyclopedia of Genes and Genomes (KEGG) and Gene Ontology (GO) terms.

### 2.5 Animal experiments

The experiments detailed here were approved by the University of Arizona IACUC (protocol 17-338). Eight-twelve (8-12) week old NOD.Cg-*Prkdc^scid^ Il2rg^tm1Wjl^* Tg(IL15)1Sz/SzJ (NSG-Tg(Hu-IL15)) (Jackson Labs, Stock No: 030890) were used for xenotransplantation of K562-luc2 tumor cells (ATCC, Manassas, VA) and human PBMCs. NSG-Tg(Hu-IL15) mice include the human IL-15 transgene allowing endogenous expression of physiological levels of human IL-15 (7.1 ± 0.3 pg/mL). Additional GvHD and human immune cell engraftment experiments were performed using standard NSG mice without human IL-15 knock-in (Jackson Labs, Stock No: 005557). On Day -2, mice were irradiated with 100cGy (Bio5 Cesium 137 Irradiator, Atomic Energy of Canada Ltd) to improve engraftment of human cells. On Day -1, mice were injected intravenously through the lateral tail vein with 1x10^7^ PBMCs either from resting or during-exercise samples. On day 0, mice were injected in the lateral tail vein with 1x10^6^ K562-luc2 cells. Bioluminescent imaging (BLI) began on day +1 post-tumor injection and repeated every 3-4 days to track tumor progression using the LargoX bioluminescence imager (Spectral Instruments Imaging, Tucson, AZ). Mice not receiving tumor or PBMCs were instead injected with an equal amount of saline. All animals were monitored daily until sacrifice. All PBMC samples were injected in duplicate or triplicate (2-3 mice, per human PBMC sample).

#### 2.5.1 K562-luc2 and PBMC preparation for injection

K562-luc2 cells were thawed from cryopreservation 48h before tumor injections and maintained at 37°C, 5% CO_2_ in Iscove’s DMEM supplemented with 10% FBS and 8µg/mL blasticidin for 2 days. On the day of tumor injections, K562-luc2 cells were removed from culture, washed three times with sterile PBS, and resuspended at a concentration of 1x10^6^ cells/200µL in sterile saline. Cryopreserved PBMCs were thawed in a 37°C water bath for two minutes or until a small ball of ice remained in solution. PBMCs were resuspended in 5mL RPMI + 10% FBS. A small aliquot (50 µL) was taken, and cells were labeled with monoclonal antibodies (CD8-VioBlue, CD14-VioGreen, CD3-FITC, CD4-PE, CD20-PerCP, CD45-APC, CD56-APC-Vio770 (Miltenyi Biotec) for surface staining prior to injection. The remaining cells were supplemented with IL-15 (0.1mg/mL) and incubated for 1 hour at 37°C, 5% CO_2_ to improve activation and recovery of NK cells functions. After incubation, PBMCs were washed three times with PBS to remove all RPMI media and then resuspended at a concentration of 10x10^6^ PBMCs/200µL sterile filtered saline for injections.

#### 2.5.2 PBMC engraftment

Human immune cell engraftment was tracked weekly. A sample of blood (50-100µL) was collected from each mouse using the submental method. 25µL of whole blood samples were stained and analyzed by flow cytometry using the following directly conjugated antibodies: CD8-VioBlue, CD14-VioGreen, CD3-FITC, CD4-PE, CD20-PerCP, CD45mouse-PE-Vio770, CD45human-APC, CD56-APC-Vio770 (Miltenyi Biotec). All antibodies, except CD45mouse, were reactive with human antigens. CD45mouse was used to exclude mouse leukocytes from the analysis.

#### 2.5.3 BLI and morbidity score

All mice were imaged every 3-4 days to monitor tumor progression on the LargoX bioluminescent imager. Briefly, mice were injected intraperitoneally with D-luciferin, potassium salt reconstituted in Dulbecco’s phosphate-buffered saline (15mg/ml) (Gold Biotechnologies, St. Louis, MO) at a concentration of 10µL/g body weight (BW). Bioluminescent images were gathered by 5-minute, 1 minute, 10 second, 5 second, or 1 second exposures. Bioluminescent data is expressed as photons/s.

On the same day of imaging, mice were weighed and scored for symptoms of xenogeneic GvHD. Scores were assessed based on the following symptoms of clinical GvHD: Skin Integrity (0-2): 0 = normal, healthy skin; 1 = Scaling of paws/tail; 2 = dehydrated, obvious areas of denuded skin; Fur Integrity (0-2): 0 = normal, fluffy, and elastic fur; 1 = mild to moderate ruffling; 2 = soiled, stiff, and rough fur; Posture (0-2): 0 = normal posture; 1 = hunching only at rest; 2 = severe hunching, sunken or distended abdomen; Activity (0-2): 0 = normal, responsive and vocal; 1 = mild to moderately decreased; 2 = unresponsive, separates from group, circling, head pressing; Weight Loss (0-2): 0 = <10%; 1 = 10% to 20%; 2 = >20%; and Diarrhea (0-1): 0 = no; 1 = yes.

Any multiple combinations of the clinical signs are indicative of a moribund condition and mice were euthanized accordingly. Because actual tumor volume cannot be determined with intravenous administration of K562, euthanasia occurred when any of the following ocurred: GvHD ≥ 8, ≥20% BW loss, hind limb paralysis and/or impaired ambulation.

### 2.6 Statistical analysis

Paired T-tests were used to detect significant differences in the total numbers of immune cell subtypes measured in blood before and after exercise. Linear mixed models with Bonferroni correction for multiple comparisons were used to detect main effects of time, group and interaction effects (time x group) for human leukocyte engraftment, GvHD, and leukemic burden (BLI). Simple survival analysis (Kaplan-Meier) was used to detect differences in overall survival, tumor-free survival, and GvHD-free survival. The percentage of starting mice that were alive and of low leukemic burden (defined as a BLI score below the 95% confidence interval of the corresponding control mice receiving only K562) were compared weekly between groups by chi-square. Significance was accepted at p<0.05. For DGE analysis by scRNAseq, fold changes in individual gene expression within each cluster was considered significant after adjusting for multiple hypothesis testing (Padj<0.05). For GSEA, the false discovery rate (FDR) was used separately for each database (GO and KEGG) to correct for multiple hypothesis testing. Given the exploratory nature of our analysis, we selected an FDR threshold of <0.25, which denotes the confidence of ‘possible’ or ‘hypothesis’, while an FDR < 0.05 denotes ‘high confidence’ or ‘statistical significance’ (13). We used the less stringent FDR for our GSEA analysis to avoid overlooking potentially meaningful changes in enriched gene sets in response to exercise.

## 3 Results

### 3.1 Exercise preferentially mobilizes effector lymphocytes with phenotypic and transcriptomic profiles associated with cytotoxicity, differentiation, migration and effector cytokine signaling

**Figure 1:**
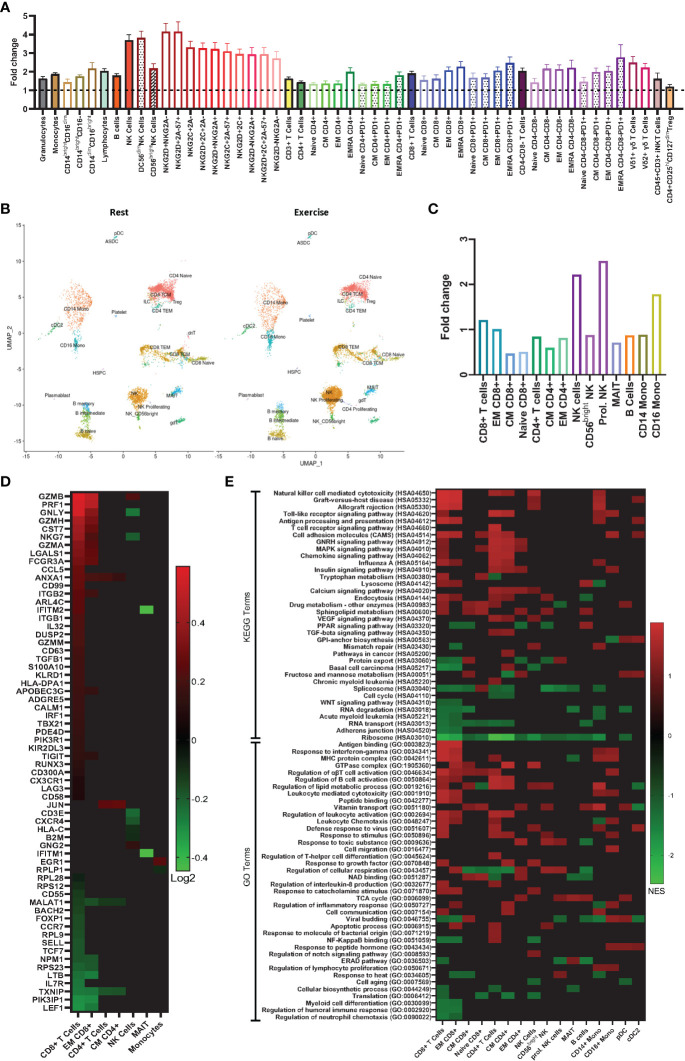
Exercise preferentially mobilizes effector lymphocytes with phenotypic and gene expression profiles associated with anti-tumor immunity. **(A)** Fold changes in absolute number of immune cell subsets in human whole blood from rest to during exercise, using the values presented in **Table 1** (exercise/rest) (n=16). **(B)** Azimuth map for human PBMCs showing the 26 clusters identified by scRNAseq at rest and during exercise. (n=3) **(C)** Fold changes in proportion of the single cells analyzed from rest to during exercise (exercise/rest)(n=3). **(D)** Heat map showing differentially expressed genes (DEG) from rest to during exercise in 7 immune cell subsets (n=3). **(E)** Gene sets annotated to Gene ontology (GO) and Kyoto Encyclopedia of Genes and Genomes (KEGG) terms that were enriched after exercise (n=3). Statistical significance: p ≤ 0.05 for DEG; and FDR ≤ 0.25 for the gene set enrichment analysis.

To enumerate circulating immune cells and determine their magnitude of change in response to exercise, we used an extensive immunophenotyping panel (**Table S1**) with 8-color flow cytometry on whole blood samples collected before and during exercise. Total cell numbers are shown in **Table 1**, with fold changes in the absolute cell number from rest to during exercise shown in **Figure 1**. We observed the archetypal exercise-induced mobilization of lymphocytes with a phenotypic profile consistent with cytotoxicity and differentiation. Consistent with previous observations (6, 14), NK-cells, γδ T-cells and CD45RA+ effector memory (EMRA) CD4+ and CD8+ T-cells were among the most exercise responsive subsets (**Figure 1A**). In contrast, suppressor cells such as CD4+ regulatory T-cells (Treg) were not significantly mobilized with exercise. A novel observation reported here is that T-cells expressing PD-1, particularly within more differentiated subsets (e.g., EMRAs) were highly responsive to the exercise stimulus. PD-1 plays an important immunoregulatory role and its expression is upregulated in activated T-cells (15), although it is associated with exhaustion and decreased cytotoxicity in CD8+ T-cells (16). Within the NK-cell population, there was a greater mobilization of CD56^dim^ cells, which are more cytotoxic, compared to the CD56^bright^ subset, which has an immunoregulatory role. We also observed a preferential mobilization of NK-cells expressing the activating receptor NKG2D relative to NK-cells expressing the inhibitory receptor NKG2A. NKG2D is a receptor that increases NK-cell expansion, cytotoxic activity, and survival, contributing to a stronger antitumor response (17). NKG2A, on the other hand, inhibits NK cell function and has become a target for cancer immune checkpoint inhibition therapy (18). This indicates that NK-cells mobilized to blood during exercise may have enhanced cytotoxic properties. There was a trend for Vδ1 T-cells to be mobilized with exercise at a greater magnitude compared to the Vδ2 subset. Vδ2 are the most prevalent subset in the circulation and possess well-established anti-tumor effector function (19). Vδ1 are a minor subset of γδ T-cells with evidence of cytotoxic, immunosuppressive and regulatory roles (20).

**Table 1:** The total number (cells/μL) of Leukocyte subsets present in peripheral blood at rest and during exercise.

Leukocyte subsets	Rest	Exercise	P Value
**Granulocytes**	2681.61 ± 1010.11	4264.69 ± 1462.87	4.28E-06
**Monocytes**	332.37 ± 68.90	623.55 ± 175.88	4.58E-07
**CD14^bright^CD16^dim^**	12.56 ± 8.34	18.32 ± 17.08	0.05
**CD14^bright^CD16-**	256.27 ± 104.02	445.38 ± 210.52	1.90E-05
**CD14^dim^CD16^bright^**	222.29 ± 85.85	501.67 ± 358.02	4.03E-03
**Lymphocytes**	1879.03 ± 282.27	3795.99 ± 1032.27	1.26E-06
**B cells**	146.24 ± 52.45	253.75 ± 90.62	4.93E-08
**NK Cells**	383.18 ± 156.75	1276.99 ± 475.70	3.26E-08
**CD56^dim^ NK cells**	383.83 ± 163.21	1292.98 ± 493.63	7.34E-07
**CD56^bright^ NK cells**	18.97 ± 10.31	38.55 ± 22.55	8.59E-04
**NKG2D+NKG2A-**	241.85 ± 124.25	866.22 ± 363.44	1.74E-06
**NKG2C+2A-57+**	23.92 ± 53.55	73.58 ± 154.10	0.09
**NKG2C+2A-**	60.07 ± 78.58	181.46 ± 217.21	0.01
**NKG2D+2C+2A-**	55.20 ± 76.69	165.85 ± 215.04	0.01
**NKG2D+NKG2A+**	127.46 ± 110.79	379.05 ± 350.37	1.54E-03
**NKG2D+2A-57+**	54.22 ± 65.95	205.53 ± 221.10	3.83E-03
**NKG2D+2C+**	68.82 ± 80.44	198.39 ± 224.69	4.35E-03
**NKG2D-NKG2A+**	6.86 ± 6.45	15.02 ± 12.23	0.01
**NKG2D+2C+2A-57+**	21.92 ± 52.39	66.14 ± 147.87	0.11
**NKG2D-NKG2A-**	26.36 ± 17.66	59.11 ± 35.63	1.55E-04
**CD3 T Cells**	1237.02 ± 247.17	2014.60 ± 712.14	8.93E-06
**CD4+ T Cells**	872.17 ± 736.74	939.69 ± 1044.46	1.48E-04
**Naive CD4+**	399.45 ± 174.48	539.83 ± 284.52	2.03E-03
**CM CD4+**	224.28 ± 118.18	305.62 ± 179.20	0.02
**EM CD4+**	87.49 ± 62.46	123.45 ± 122.32	0.15
**EMRA CD4+**	33.36 ± 26.80	75.54 ± 89.82	0.06
**Naive T CD4+PD1+**	304.47 ± 156.05	412.60 ± 255.23	0.01
**CM T CD4+PD1+**	167.76 ± 66.60	235.79 ± 145.97	0.02
**EM T CD4+PD1+**	72.65 ± 49.18	109.68 ± 122.17	0.12
**EMRA T CD4+PD1+**	22.24 ± 16.47	47.36 ± 43.81	0.02
**CD8+ T Cells**	393.07 ± 88.21	756.73 ± 331.33	2.05E-05
**Naive CD8+**	82.41 ± 43.04	128.24 ± 86.64	0.02
**CM CD8+**	47.99 ± 27.01	86.82 ± 96.71	0.06
**EM CD8+**	137.64 ± 63.12	302.32 ± 246.15	4.96E-03
**EMRA CD8+**	128.61 ± 91.00	259.46 ± 185.51	4.74E-04
**Naive T CD8+PD1+**	47.29 ± 49.24	77.30 ± 88.14	0.07
**CM T CD8+PD1+**	27.82 ± 16.14	47.88 ± 46.93	0.05
**EM T CD8+PD1+**	75.95 ± 40.51	162.48 ± 124.07	3.35E-03
**EMRA T CD8+PD1+**	61.93 ± 72.20	123.65 ± 159.67	0.03
**CD4-CD8- T Cells**	179.86 ± 94.54	440.26 ± 189.65	6.71E-06
**Naive CD4-CD8-**	11.92 ± 13.19	13.72 ± 18.59	0.70
**CM CD4-CD8-**	26.82 ± 27.78	55.63 ± 68.68	0.03
**EM CD4-CD8-**	38.23 ± 42.97	81.39 ± 95.66	0.01
**EMRA CD4-CD8-**	24.63 ± 32.57	54.57 ± 68.92	0.01
**Naive CD4-CD8-PD1+**	9.82 ± 13.58	10.97 ± 19.21	0.79
**CM CD4-CD8-PD1+**	17.48 ± 21.44	34.24 ± 48.21	0.04
**EM CD4-CD8-PD1+**	15.02 ± 18.96	28.18 ± 36.32	0.02
**EMRA CD4-CD8-PD1+**	9.52 ± 16.48	21.54 ± 41.90	0.10
**Vδ1+ γδ T Cells**	20.96 ± 20.38	52.99 ± 55.25	0.01
**Vδ2+ γδ T Cells**	91.74 ± 75.79	183.59 ± 157.15	1.03E-03
**CD45+CD3+iNKT Cells**	15.99 ± 14.21	120.99 ± 384.92	0.30
**CD4+CD25^hi^CD127^dim^ Treg**	28.10 ± 23.53	33.72 ± 33.98	0.22

P values are displayed in the right column. Significance P ≤ 0.05.

To investigate the effects of exercise on the transcriptome within PBMCs, we performed single-cell RNA sequencing (scRNAseq) and identified 26 cell clusters in accordance with the Azimuth map for human PBMCs (**Figure 1B**) following the methods described by Hao and Hao et al. (21). DGE between rest and exercise-mobilized PBMCs were observed in 7 cell types (**Figure 1D**). A description of each DGE in accordance with GeneCards^®^ is provided in **Table S2**. We found a greater number of DGEs in response to exercise within the CD8+ T-cell subset. Genes associated with increased cytotoxicity (GZMB, GAMH, PRF1- (22)), IFN-γ production (ANXA1- (23)), cellular adhesion and migration (ITGB1- (24), ITGB2, CCL5- (25)) and anti-tumor activity (HLA-DPA1, NKG7- (26)) were enriched, while genes encoding proteins that suppress immune function and differentiation (MALAT1- (27), TXNIP - (28), FOXP1- (29)) were downregulated. The majority of these DGEs occurred within the EM subset of CD8+ T-cells. These results re-enforce the concept that exercise preferentially mobilizes effector lymphocytes with phenotypic and transcriptomic profiles associated with cytotoxicity, differentiation, migration, and effector cytokine signaling.

### 3.2 scRNAseq reveals enrichment of gene sets associated with anti-tumor activity in exercise mobilized lymphocytes

**Figure 2:**
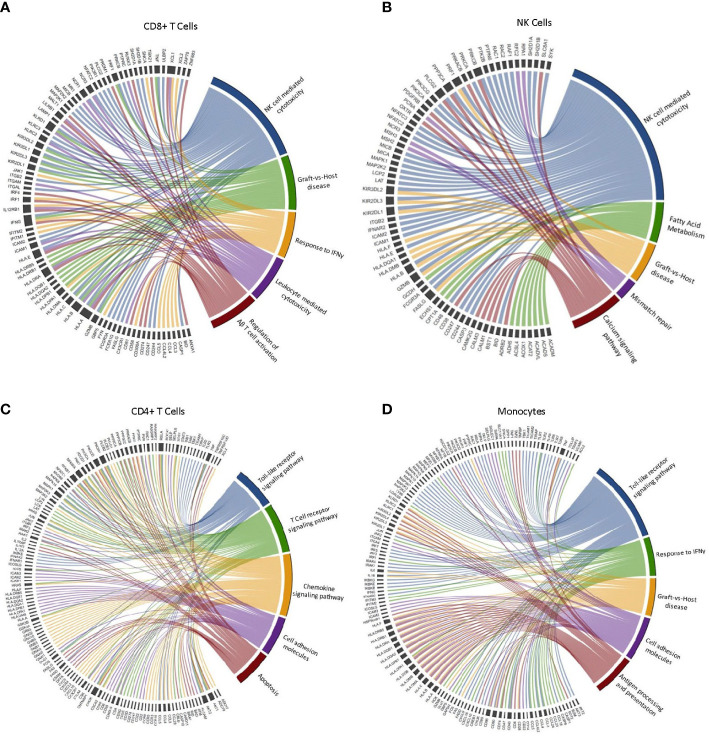
Leading-edge genes driving the enrichment of selected GO and KEGG terms in CD4+ T-cells, CD8+ T-cells, NK-cells and monocytes after exercise. Chord diagram displaying the leading-edge genes driving the enrichment of terms associated with cytotoxicity and anti-tumor immune activity in **(A)** CD8+ T cells, **(B)** NK cells, **(C)** CD4+ T cells, and **(D)** Monocytes. (n=3).

To identify biological processes associated with our DGE data, we performed functional annotation and enrichment analysis using both GO and KEGG terms (**Figure 1E**). The greatest number of gene set enrichments were found in CD8+ T-cells, with an upregulation of gene sets associated with cytotoxicity, cellular adhesion and migration, cellular proliferation, and anti-tumor activity. These were mostly driven by the EM population, with a smaller contribution of central memory (CM) and naïve CD8+ T-cells. We observed an upregulation of gene sets involved in GvHD and allograft rejection within CD8+ T-cells, NK-cells, monocytes, and dendritic cells. This was driven mostly by HLA and KIR genes (**Figures 2A, B**), which are also involved in GvL effects (30). Gene sets involved in MAPK signaling were enriched in total and naïve CD8+ T-cells, as well as total, CM, and EM CD4+ T-cells. MAPK pathways play an important role in the regulation of T-cell proliferation and differentiation, although, the signaling cascade might differ between naïve and antigen-experienced T-cells (31). Gene sets involved in calcium and insulin signaling were enriched in CD4+ T-cells and CD16+ monocytes, while the sphingolipid metabolism gene set was enriched in most lymphocytes and downregulated in plasmacytoid dendritic cells (pDCs). Sphingolipids are a major component of cellular membranes, and their metabolism is associated with T-cell viability and function (32). Interestingly, gene sets involved in protein synthesis (e.g., ‘ribosome’ and ‘translation’) were downregulated in almost all cell types in response to exercise (**Figure 2**). Gene sets included in the terms ‘defense response to virus’ and ‘antigen processing and presentation’ were enriched in some lymphocytes (CD8+, CD4+, and NK-Cells) and antigen presenting cells (APCs; CD14+ monocytes, and cDC2s). Gene sets associated with cellular respiration were enriched in EM CD4+ and CD8+ T-cells, and NK-cells, but downregulated in naïve and CM T-cells, which might be explained by the higher ATP demand of effector and cytotoxic cells (33). Similarly, Gene sets associated with tricarboxylic acid (TCA) cycle were enriched on CM CD4+ T-cells, proliferating NK-cells, MAIT, B-cells, and pDCs.

We selected five GO/KEGG terms associated with antitumor immune activity to display the leading-edge genes driving the enrichment within each major cell type (**Figure 2**). Interestingly, we found variability in the leading-edge genes enriching the selected terms across all cell types, with CD8+ T-cells presenting a higher number of the KIR and HLA family genes enriching NK-cell mediated cytotoxicity, when compared to NK-cells. However, there is a high level of overlap in gene sets enriching the selected terms within the same cell type. For example, GvHD and NK-cell mediated cytotoxicity are mostly driven by the same genes. Similarities can also be seen in the leading-edge genes driving enrichment of the ‘response to IFN-γ’ and ‘Toll-like receptor signaling’ terms. Overall, our results indicate that PBMCs mobilized to blood during exercise are more alloreactive and cytotoxic and should, therefore, have greater potential to elicit GvL effects if used as a DLI.

### 3.3 Exercise-mobilized PBMCs extend survival and reduce leukemic burden in xenogeneic mice

**Figure 3:**
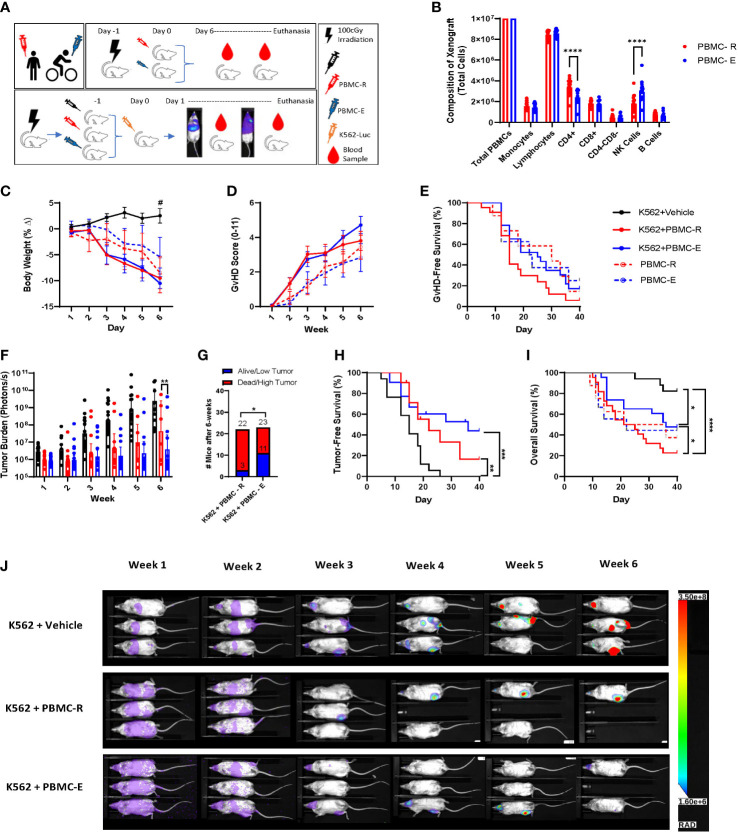
Exercise-mobilized PBMCs extend survival and reduce leukemic burden in xenogeneic mice. To determine the GvL effect of the PBMCs mobilized by exercise, NSG-IL15 mice were injected with PBMCs (10x10^6^) collected either at rest (PBMC-R) or during (PBMC-E) exercise and challenged with 1x10^6^ luciferase tagged human chronic myeloid leukemia cells (K562-luc). **(A)** Illustration of the experiment design. **(B)** composition of the PBMC-R and PBMC-E xenografts. **(C)** body weight and **(D)** GvHD score measured twice weekly. **(E)** GvHD-free survival determined using a composite score of ≥4. **(F)** tumor burden measured twice weekly *via* BLI quantification (photons/s). **(G)** low tumor burden was defined as a BLI score <95% confidence interval of K562+vehicle at the corresponding time point. **(H)** Tumor-free survival. Mice were considered tumor-free until the BLI score surpassed the individual value recorded on day 1. **(I)** Overall survival of leukemia-bearing mice that received vehicle (K562+vehicle, solid black line), resting PBMCs (K562+PBMC-R, solid red line) or exercise PBMCs (K562+PBMC-E, solid blue line); and in non-tumor bearing mice that received resting (PBMC-R, dashed red line) or exercise (PBMC-E, dashed blue line) PBMCs. **(J)** representative bioluminescence image of leukeamia-bearing mice that received vehicle (top row), resting PBMCs (middle row), or exercise PBMC (bottom row), showking BLI intensity on a scale from low (purple) to high (red). *P ≤ 0.05, **P ≤ 0.01, ***P ≤ 0.001, ****P ≤ 0.0001, ^#^Different from K562+Vehicle P ≤ 0.05. (n=20-23 mice/group).

**Figure 4:**
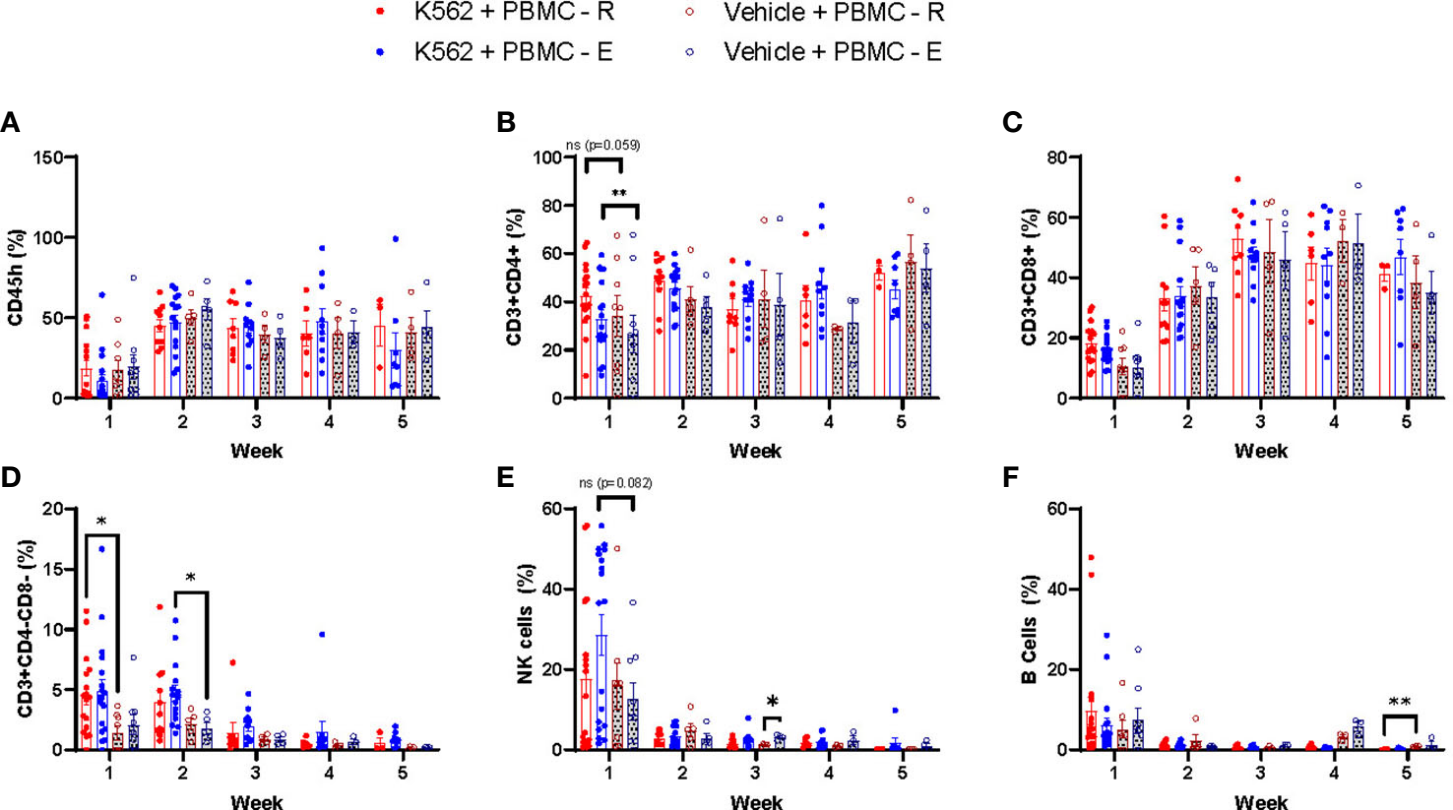
Human leukemia expands effector lymphocytes in xenogeneic mice receiving exercise-mobilized but not resting PBMCs. To track the human cells engraftment, mice blood was collected weekly, being the first samples collected 7 days after the PBMC injections, and whole blood was labeled with monoclonal antibodies for the analysis of the following cell types: **(A)** CD45h, **(B)** CD4+ T cells, **(C)** CD8+ T cells, **(D)** DN T cells **(E)** NK cells, and **(F)** B cells in leukemia-bearing mice that received resting PBMCs (K562+PBMC-R, solid red dot) or exercise PBMCs (K562+PBMC-E, solid blue dot); and in non-tumor bearing mice that received resting (PBMC-R, open red dot) or exercise (PBMC-E, open blue dot) PBMCs. *P ≤ 0.05, **P ≤ 0.01. (n=11-23 mice/group).

We found that PBMCs mobilized to blood during exercise have surface phenotypes and transcriptomes associated with anti-tumor immunity, including cytotoxicity, migration/chemotaxis, antigen binding, cytokine responsiveness and alloreactivity (e.g. GvHD/GvL). Our next step was to determine if DLI comprised of exercise-mobilized PBMCs would evoke greater GvL effects *in vivo* without exacerbating xenogeneic GvHD. We used the highly immunodeficient NSG mouse strain coupled with a human IL-15 knock-in to support persistence of adoptively transferred NK-cells as well as T-cells. Mice received resting (PBMC-R) or exercise-mobilized (PBMC-E) PBMCs on day -1, while a set of mice were injected with vehicle (saline). Mice were then injected with a luciferase-tagged human chronic myeloid leukemia (CML) cell line (K562-luc2) on day 1. To track GvHD in the absence of tumor, sets of mice were injected with PBMC-R or PBMC-E only (**Figure 3A**). All mice received an equal number of 1x10^7^ PBMCs, however, as expected, exercise altered the composition of cells in the xenograft. Mice injected with exercise-mobilized PBMCs received significantly less CD4+ T-cells and more NK-cells (**Figure 3B**). To track human cell engraftment, murine blood was collected weekly and assessed for the presence of major human lymphocyte populations by flow cytometry (**Table S1**). No major difference in human immune cell engraftment was found between the groups (**Figure 4**). Mice achieved ~50% human PBMC engraftment by week 2 with no differences between the groups (**Figure 4A**). Similarly, engraftment of lymphocyte subtypes did not differ significantly between mice that received resting or exercise-mobilized PBMCs. However, at week 1, NK-cell frequencies trended higher in the K562+PBMC-E group compared to mice that received vehicle+PBMC-E. There was also significantly more CD4+ T-cells at week 1, and CD3+/CD4-/CD8- ‘double-negative’ (DN) T-cells at week 2 when comparing these groups, indicating a potential interaction between K562 stimulation and the expansion/engraftment of exercise-mobilized PBMCs *in vivo*.

All mice that received PBMCs had a greater reduction in BW compared to mice receiving K562+vehicle due to xenogeneic GvHD (**Figure 3C**); however, no difference in GvHD score or GvHD-free survival (using a composite score of ≥4) was observed between groups (**Figures 3D, E**). As expected, mice that did not receive PBMCs and were injected only with K562+vehicle had the highest tumor burden (**Figure 3F**), demonstrating that the adoptively transferred PBMCs helped limit leukemic progression *in vivo*. By week 3, mice injected with exercise-mobilized PBMCs started to display lower leukemic burden compared to the group injected with resting PBMCs, which reached statistical significance at week 6 (**Figures 3F, J**). Also, 11 of 23 mice in the K562+PBMC-E group were alive and/or had low tumor (defined as a BLI score <95% confidence interval of K562+vehicle at the corresponding time point) at week 6, compared to only 3 of 22 mice in the K562+PBMC-R group, (**Figure 3G**). which was reflected by improved, but not significant, tumor-free survival (**Figure 3H**) and significantly higher overall survival (**Figures 3I, J**). Finally, we used the standard NSG mouse model, which is known to have slower GvHD manifestation kinetics, to see if there were differences in human cell engraftment and xenogeneic GvHD between resting and exercise-mobilized PBMCs in a less severe model. Similar to the NSG-Tg (Hu-IL15) model, we found no differences in overall survival, GvHD score, GvHD survival, body weight, or engraftment with human immune cells (**Supplemental Figure 1**). Our results confirmed our hypothesis that DLI collected during exercise would enhance the GvL effect without intensifying GvHD as we observed a slower rate of leukemic progression, improved overall survival, and a trend toward improved GvHD-free survival in the mice that received exercise mobilized PBMCs.

## 4 Discussion

Every bout of dynamic exercise evokes the mobilization and redistribution of effector lymphocytes, which may play a role in improving cancer immune surveillance in habitual exercisers. This is the first study, to our knowledge, to provide a comprehensive single cell transcriptomic analysis of human mononuclear cells mobilized to blood with exercise, and the first to assess anti-leukemic effects of these mobilized cells in a xenogeneic mouse model of human leukemia. We found that effector lymphocytes mobilized with exercise, particularly effector-memory CD8+ T-cells and NK-cells, have differentially expressed genes and enriched gene sets associated with anti-tumor activity such as cytotoxicity, migration/chemotaxis, antigen binding, cytokine responsiveness and alloreactivity (e.g. graft-versus-host/leukemia). Additionally, when used as a donor lymphocyte infusion (DLI), exercise-mobilized cells extended survival and reduced K562 leukemic burden in xenogeneic mice without exacerbating GvHD.

Single exercise bouts are known to preferentially mobilize effector subsets of lymphocytes and monocytes to the blood compartment. NK-cells, γδ T-cells, and CD8+ T-cells are among the most exercise-responsive lymphocytes, while CD16+ ‘non-classical’ monocytes are mobilized in relatively greater numbers than their ‘classical’ counterparts (14). We performed extended flow cytometry to enumerate multiple subsets of human lymphocytes and monocytes before and after exercise. Confirming previous findings (1), we saw a step-wise preferential mobilization of CD8+ T-cells in accordance with differentiation status and that EMRA subsets of CD4+ T-cells were preferentially mobilized over their less differentiated CD4+ counterparts. We also noted that subsets of CD8+ T-cells expressing the immune checkpoint and marker of T-cell exhaustion, PD-1 (34), are highly responsive to exercise. This may have important implications for studies attempting to combine exercise with immune checkpoint inhibitors to enhance the therapeutic response (35). Suppressor cells such as CD4+ T-regs are not significantly mobilized with exercise, thus increasing the ratio of effector to suppressor cells among lymphocytes in response to exercise, which is considered beneficial for adoptively transferred cell therapies (3).

We have provided the most comprehensive single cell gene expression profile of exercise-mobilized lymphocytes and monocytes in humans to date. Clustering cells by their transcriptomic signature in accordance with the Azimuth PBMC reference atlas, and using a targeted gene expression panel curated for human immunology, allowed us to identify over 9000 genes and 64 differentially expressed genes in 26 discrete subsets of lymphocytes and monocytes (21). Consistent with the premise that exercise preferentially mobilizes lymphocytes with enhanced cytotoxic potential, we found an upregulation of genes coding for cytolytic granules (e.g. granzyme A, B, H and M; perforin, NKG7) within CD8+ T-cells, an effect that was largely driven by gene expression changes within the CD8+ EM subset. Genes coding for proteins involved in the regulation of T-cell and NK-cell cytotoxicity (e.g. CST7, FCGR3A) (36) were also enriched within CD8+ EM cells, as were genes coding for proteins involved in chemotaxis and migration (e.g. CCL5, CD99) (25), as well as activation and apoptosis (e.g. ANXA1, LGALS1) (23). Interestingly, despite NK-cells being the most ‘exercise-responsive’ lymphocyte subset, fewer gene expression changes were seen in NK-cells compared to CD8+ T-cells. This could be due to greater heterogeneity among the CD8+ T-cell compartment and the propensity for exercise to mobilize only the most differentiated and cytotoxic CD8+ subsets. We also found that expression levels of certain genes changed divergently with exercise dependent on cell type. For instance, NKG7 gene expression was enriched with exercise in CD8+ T-cells but downregulated in NK-cells. This could have implications for cancer patients on immune checkpoint inhibitors, as low NKG7 expression in CD8+ T-cells has been shown to predict poor responders to anti PD-1 therapy (37). Furthermore, NKG7 expression in CD8+ T-cells regulates the translocation of CD107a to the membrane enhancing their ability to kill malignant cells (26). It is possible that exercise may increase the cytotoxic effects of CD8+ T-cells directly through NKG7 transcription and the concomitant upregulation of cytolytic granules and calcium release, but this remains to be determined. Conversely, genes involved in the development of suppressor functions (e.g. TCF1, LEF1) (38), glucose metabolism, and inhibition and cell quiescence (e.g. TXNIP, FOXP1) (32,43) were downregulated, indicating that exercise-mobilized CD8+ T-cells have increased metabolic activity and are more permissive upon activation.

Functional annotation and enrichment analyses revealed an upregulation of gene sets associated with anti-tumor immunity, especially within subsets of CD8+ T-cells and to a lesser extent CD4+ T-cells. These included GO and KEGG terms related to antigen binding and processing, cytotoxicity, alloreactivity (e.g. GvHD), adhesion/migration, cytokine/chemokine responsiveness and toll-like receptor signaling. Similar terms were enriched among the APCs (e.g. monocyte and dendritic cell subsets). Interestingly, alloreactivity/GvHD was enriched in multiple cell types after exercise including total CD8+ T-cells, EM CD8+ T-cells, NK-cells, classical monocytes and cDC2 cells. While these findings indicate that exercise mobilized lymphocytes and APCs are more alloreactive and could increase risk of GvHD in the allogeneic adoptive cell therapy setting, it is important to note that the GvL effects of these cells is also likely to be greater (39, 40). We also found a large number of gene sets enriched within exercise responsive monocytes. Antigen presentation/processing, cytotoxicity, cytokine responsiveness, fatty acid metabolism, vitamin transport and defense response to virus were enriched in ‘classical’ monocytes after exercise. The preferentially mobilized ‘non-classical’ monocytes had an upregulation of gene sets involved in T-cell activation and proliferation, migration/chemotaxis and responsiveness to cytokines, carbohydrates, insulin and hormones. Changes within pDCs were largely related to macronutrient metabolism. Overall, this detailed single cell transcriptomic analysis has revealed exercise mobilized monocytes and lymphocytes (particularly CD8+ T-cells) to have increased gene expression profiles associated with enhanced cytotoxic, migration, activation, cytokine responsiveness, and alloreactive functions. This indicates that exercise-mobilized lymphocytes would be highly effective as an allogeneic cell therapeutic product (e.g. DLI) for the treatment of hematologic malignancies, providing that exacerbation of GvHD is not a limiting factor.

To test whether exercise mobilized lymphocytes have the potential to improve DLI anti-leukemic effects, we engrafted xenogeneic mice with PBMCs collected before and during exercise prior to challenging the mice with K562 cells. By the end of the experiment, more mice that received exercise-mobilized cells (~48%) were alive and had lower tumor burden than mice that received resting cells (~14%) from the same donors. Encouragingly, GvHD effects were not different between NSG-Tg (Hu-IL15) or standard NSG mice receiving resting or exercise-mobilized lymphocytes in the absence of tumor. It is possible that the altered composition of the xenografts evoked by exercise are responsible for the enhanced GvL effects without concomitant increases in GvHD. As expected, exercise lowered the total number of CD4+ T-cells and increased the number of NK-cells without affecting the total number of other cell types in the graft (1). CD4+ T-cells are known to be highly alloreactive and can cause severe GvHD (41), whereas NK-cells are minimally alloreactive and can exert strong GvL effects (42), particularly against the K562 cell line which is HLA-deficient and a known NK-cell sensitive target (43). Furthermore, increased NK-cell cytotoxicity after exercise is associated with an elevated ratio of activating to inhibitory receptor expression particularly if the target cells express ligands for NK-cell activating receptors (44). The increased GvL effects seen in xenogeneic mice receiving exercise-mobilized DLI is most likely driven by the greater numbers of NK-cells in the graft, as we have shown previously that exercise-mobilized lymphocytes have similar cytotoxic activity against K562 cells *in vitro* after adjusting for NK-cell numbers (44). It is possible, however, that some of our observed transcriptomic changes could contribute to increased cytotoxicity of exercise-mobilized NK-cells *in vivo*, but this requires further investigation.

The human IL-15 knock in strain of the highly immunodeficient NSG mouse was used here to support persistence of the exercise-mobilized NK-cells after adoptive transfer (45). This is particularly important as NK-cell infiltration has been purported to play a mechanistic role in reducing B16 melanoma burden in mice exposed to voluntary wheel running (8), while IL-15 responsive CD8+ T-cells were recently shown to suppress tumor growth in a mouse exercise model of pancreatic ductal adenocarcinoma (46). Our data indicate that NK-cells are also involved in the anti-leukemic effects of exercise, as K562 challenge tended to increase the numbers of human NK-cells and significantly increased ‘double negative’ (DN) T-cells (which largely consist of γδ T-cells and MAIT cells) in mouse peripheral blood within the first 1-2 weeks, but only if they received exercise mobilized lymphocytes. Although we did not observe differences in IL15R gene expression between resting and exercise mobilized NK-cells, the preferential mobilization of NKG2D+ NK-cells by exercise may have provided a greater number of IL-15 responsive NK-cells in the exercise mobilized grafts, as NK-cells bearing this receptor are more responsive to IL-15 stimulation (47). We have previously shown that exercise mobilized T-cells and γδ T-cells proliferate better *ex vivo* in response to tumor antigen (e.g. WT1, PRAME) peptide loaded dendritic cells and phosphate antigens, respectively (48, 49). Moreover, γδ T-cells expanded after exercise have heightened expression of several activating receptors involved in anti-tumor immunity and are superior killers of multiple hematologic cancer cell lines *in vitro* (49). This indicates that exercise mobilized NK-cells and DN T-cells are proliferating/persisting more readily in response to K562 challenge compared to their counterparts in resting blood, although future studies are required to determine if they play a mechanistic role in reducing leukemic burden after adoptive transfer. It will also be important to determine if exercise-mobilized lymphocytes can curtail growth of pre-established leukemias and to evaluate the response across different hematologic malignancies. Finally, future studies should determine if the gene expression changes and/or graft composition shifts reported herein play a mechanistic role in enhancing the GvL effects of exercise-mobilized lymphocytes.

We conclude that exercise in humans mobilizes effector lymphocytes with an anti-tumor transcriptomic profile and that their use as a DLI extends survival and enhances the GvL effect without exacerbating GvHD in human leukemia bearing xenogeneic mice. These findings provide mechanistic insight into how human lymphocyte and monocyte redistribution with exercise might contribute to the elimination of premalignant and malignant cells in the host, whilst highlighting the potential for exercise-mobilized lymphocytes to be used as an allogeneic cell therapy to effectively and economically increase the GvL effects without exacerbating GvHD.

## Data availability statement

The data presented in the study are deposited in the GEO repository, accession number GSE212740. Data can also be found at the link: https://www.ncbi.nlm.nih.gov/geo/query/acc.cgi?acc=GSE212740.

## Ethics statement

The studies involving human participants were reviewed and approved by Human Subjects Protection Program at the University of Arizona (#1801161041). The patients/participants provided their written informed consent to participate in this study. The animal study was reviewed and approved by University of Arizona IACUC (protocol 17-338).

## Author contributions

Conception or design of the work – DD, EK, and RS. Data collection – HB, DD, GN, FB, KS, TZ, PM, and MS. Data analysis and interpretation.– HB, DD, FB, BL, EK, and RS. Drafting the article – HB, RS, and EK. Critical revision of the article - HB, DD, GN, FB, KS, TZ, PM, MS, BL, EL, MG, EK, and RS. All authors contributed to the article and approved the submitted version.

## Supplementary material

Supplementary Figure 1 Exercise-mobilized PBMCs have no effect on survival, GvHD or human immune cell engraftment in non-tumor bearing standard NSG mice. To determine the GvHD effect of the PBMCs mobilized by exercise in a less severe model, standard NSG mice were injected with PBMCs (10x10^6^) collected either at rest (PBMC-R) or during (PBMC-E) exercise. **(A)** Illustration of the experimental design. **(B)** Overall survival. **(C)** GvHD score measured twice weekly. **(D)** GvHD-free survival determined using a composite score of ≥4. **(E)** body weight. To track the human immune cell engraftment, mouse blood was collected weekly starting at Day+7 and whole blood was labeled with monoclonal antibodies to enumerate the following human cell types: **(F)** CD45h, **(G)** CD4+ T cells, **(H)** CD8+ T cells, **(I)** DN T cells **(J)** NK cells, and (K) B cells. There were no significant differences between PBMC-R and PBMC-E (p>0.05). (n=11 mice/group).

## References

[1] Simpson RJ Bigley AB Agha N Hanley PJ Bollard CM. Mobilizing immune cells with exercise for cancer immunotherapy. Exerc Sport Sci Rev (2017) 45:163–72. doi: 10.1249/JES.0000000000000114 PMC6814300 28418996

[2] Ashcraft KA Warner AB Jones LW Dewhirst MW. Exercise as adjunct therapy in cancer. Semin Radiat Oncol (2019) 29:16–24. doi: 10.1016/j.semradonc.2018.10.001 30573180 PMC6656408

[3] Gustafson MP Wheatley-Guy CM Rosenthal AC Gastineau DA Katsanis E Johnson BD. Exercise and the immune system: Taking steps to improve responses to cancer immunotherapy. J Immunother Cancer (2021) 9:e001872. doi: 10.1136/jitc-2020-001872 34215686 PMC8256759

[4] Brown JC Winters-Stone K Lee A Schmitz KH. Cancer, physical activity, and exercise. Compr Physiol (2012) 2:2775–809. doi: 10.1002/cphy.c120005 PMC4122430 23720265

[5] Rodríguez-Cañamero S Cobo-Cuenca AI Carmona-Torres JM Pozuelo-Carrascosa DP Santacruz-Salas E Rabanales-Sotos JA. Impact of physical exercise in advanced-stage cancer patients: Systematic review and meta-analysis. Cancer Med (2022) 11(19):3714–27. doi: 10.1002/cam4.4746 PMC9554454 35411694

[6] Simpson RJ Boßlau TK Weyh C Niemiro GM Batatinha H Smith KA. Exercise and adrenergic regulation of immunity. Brain Behav Immun (2021) 97:303–18. doi: 10.1016/j.bbi.2021.07.010 34302965

[7] Emery A Moore S Turner JE Campbell JP. Reframing how physical activity reduces the incidence of clinically-diagnosed cancers: Appraising exercise-induced immuno-modulation as an integral mechanism. Front Oncol (2022) 12:788113. doi: 10.3389/fonc.2022.788113 35359426 PMC8964011

[8] Pedersen L Idorn M Olofsson GH Lauenborg B Nookaew I Hansen RH. Voluntary running suppresses tumor growth through epinephrine- and IL-6-Dependent NK cell mobilization and redistribution. Cell Metab (2016) 23:554–62. doi: 10.1016/j.cmet.2016.01.011 26895752

[9] Rundqvist H Veliça P Barbieri L Gameiro PA Bargiela D Gojkovic M. Cytotoxic T-cells mediate exercise-induced reductions in tumor growth. eLife (2020) 9:e59996. doi: 10.7554/eLife.59996 33095157 PMC7584454

[10] Chang X Zang X Xia CQ. New strategies of DLI in the management of relapse of hematological malignancies after allogeneic hematopoietic SCT. Bone Marrow Transplant (2016) 51:324–32. doi: 10.1038/bmt.2015.288 26595077

[11] Frey NV Porter DL. Graft-versus-host disease after donor leukocyte infusions: presentation and management. Best Pract Res Clin Haematol (2008) 21:205–22. doi: 10.1016/j.beha.2008.02.007 PMC2504712 18503987

[12] Balady GJ Chaitman B Driscoll D Foster C Froelicher E Gordon N. Recommendations for cardiovascular screening, staffing, and emergency policies at health/fitness facilities. Circulation (1998) 97:2283–93. doi: 10.1161/01.CIR.97.22.2283 9631884

[13] Zhang K Cui S Chang S Zhang L Wang J. I-GSEA4GWAS: a web server for identification of pathways/gene sets associated with traits by applying an improved gene set enrichment analysis to genome-wide association study. Nucleic Acids Res (2010) 38:W90–95. doi: 10.1093/nar/gkq324 PMC2896119 20435672

[14] Graff RM Kunz HE Agha NH Baker FL Laughlin M Bigley AB. β2-adrenergic receptor signaling mediates the preferential mobilization of differentiated subsets of CD8+ T-cells, NK-cells and non-classical monocytes in response to acute exercise in humans. Brain Behav Immun (2018) 74:143–53. doi: 10.1016/j.bbi.2018.08.017 PMC12977291 30172948

[15] Jubel JM Barbati ZR Burger C Wirtz DC Schildberg FA. The role of PD-1 in acute and chronic infection. Front Immunol (2020) 11:487. doi: 10.3389/fimmu.2020.00487 32265932 PMC7105608

[16] Ma J Zheng B Goswami S Meng L Zhang D Cao C. PD1Hi CD8+ T cells correlate with exhausted signature and poor clinical outcome in hepatocellular carcinoma. J Immunother Cancer (2019) 7:331. doi: 10.1186/s40425-019-0814-7 31783783 PMC6884778

[17] Frazao A Rethacker L Messaoudene M Avril M-F Toubert A Dulphy N. NKG2D/NKG2-ligand pathway offers new opportunities in cancer treatment. Front Immunol (2019) 10:661. doi: 10.3389/fimmu.2019.00661 30984204 PMC6449444

[18] Creelan BC Antonia SJ. The NKG2A immune checkpoint - a new direction in cancer immunotherapy. Nat Rev Clin Oncol (2019) 16:277–8. doi: 10.1038/s41571-019-0182-8 30824813

[19] Ryan PL Sumaria N Holland CJ Bradford CM Izotova N Grandjean CL. Heterogeneous yet stable Vδ2(+) T-cell profiles define distinct cytotoxic effector potentials in healthy human individuals. Proc Natl Acad Sci U S A (2016) 113:14378–83. doi: 10.1073/pnas.1611098113 PMC5167212 27911793

[20] Siegers GM Lamb LS. Cytotoxic and regulatory properties of circulating Vδ1+ γδ T cells: a new player on the cell therapy field? Mol Ther J Am Soc Gene Ther (2014) 22:1416–22. doi: 10.1038/mt.2014.104 PMC4435582 24895997

[21] Hao Y Hao S Andersen-Nissen E Mauck WM Zheng S Butler A. Integrated analysis of multimodal single-cell data. Cell (2021) 184:3573–3587.e29. doi: 10.1016/j.cell.2021.04.048 34062119 PMC8238499

[22] Roufas C Chasiotis D Makris A Efstathiades C Dimopoulos C Zaravinos A. The expression and prognostic impact of immune cytolytic activity-related markers in human malignancies: A comprehensive meta-analysis. Front Oncol (2018) 8:27. doi: 10.3389/fonc.2018.00027 29515971 PMC5826382

[23] Yang YH Song W Deane JA Kao W Ooi JD Ngo D. Deficiency of annexin A1 in CD4+ T cells exacerbates T cell-dependent inflammation. J Immunol Baltim Md (1950) 2013:190:997–1007. doi: 10.4049/jimmunol.1202236 23267026

[24] Morrison VL MacPherson M Savinko T Lek HS Prescott A Fagerholm SC. The β2 integrin-kindlin-3 interaction is essential for T-cell homing but dispensable for T-cell activation *in vivo* . Blood (2013) 122:1428–36. doi: 10.1182/blood-2013-02-484998 PMC3750339 23823319

[25] Murooka TT Rahbar R Platanias LC Fish EN. CCL5-mediated T-cell chemotaxis involves the initiation of mRNA translation through mTOR/4E-BP1. Blood (2008) 111:4892–901. doi: 10.1182/blood-2007-11-125039 PMC2384123 18337562

[26] Ng SS De Labastida Rivera F Yan J Corvino D Das I Zhang P. The NK cell granule protein NKG7 regulates cytotoxic granule exocytosis and inflammation. Nat Immunol (2020) 21:1205–18. doi: 10.1038/s41590-020-0758-6 PMC7965849 32839608

[27] Hewitson JP West KA James KR Rani GF Dey N Romano A. Malat1 suppresses immunity to infection through promoting expression of maf and IL-10 in Th cells. J Immunol Baltim Md (1950) 2020:204:2949–60. doi: 10.4049/jimmunol.1900940 PMC7231852 32321759

[28] Muri J Thut H Kopf M. The thioredoxin-1 inhibitor txnip restrains effector T-cell and germinal center b-cell expansion. Eur J Immunol (2021) 51:115–24. doi: 10.1002/eji.202048851 32902872

[29] Wei H Geng J Shi B Liu Z Wang Y-H Stevens AC. Cutting edge: Foxp1 controls naive CD8+ T cell quiescence by simultaneously repressing key pathways in cellular metabolism and cell cycle progression. J Immunol Baltim Md (1950) 2016:196:3537–41. doi: 10.4049/jimmunol.1501896 PMC4868629 27001958

[30] Gao F Ye Y Gao Y Huang H Zhao Y. Influence of KIR and NK cell reconstitution in the outcomes of hematopoietic stem cell transplantation. Front Immunol (2020) 11:2022. doi: 10.3389/fimmu.2020.02022 32983145 PMC7493622

[31] Adachi K Davis MM. T-Cell receptor ligation induces distinct signaling pathways in naive vs. antigen-experienced T cells. Proc Natl Acad Sci U S A (2011) 108:1549–54. doi: 10.1073/pnas.1017340108 PMC3029746 21205892

[32] Avota E de Lira MN Schneider-Schaulies S. Sphingomyelin breakdown in T cells: Role of membrane compartmentalization in T cell signaling and interference by a pathogen. Front Cell Dev Biol (2019) 7:152. doi: 10.3389/fcell.2019.00152 31457008 PMC6700246

[33] Batatinha HAP Biondo LA Lira FS Castell LM Rosa-Neto JC. Nutrients, immune system, and exercise: Where will it take us? Nutr Burbank Los Angel Cty Calif (2019) 61:151–6. doi: 10.1016/j.nut.2018.09.019 30711864

[34] Simon S Labarriere N. PD-1 expression on tumor-specific T cells: Friend or foe for immunotherapy? Oncoimmunology (2017) 7:e1364828. doi: 10.1080/2162402X.2017.1364828 29296515 PMC5739549

[35] Shaver AL Sharma S Nikita N Lefler DS Basu-Mallick A Johnson JM. The effects of physical activity on cancer patients undergoing treatment with immune checkpoint inhibitors: A scoping review. Cancers (2021) 13:6364. doi: 10.3390/cancers13246364 34944984 PMC8699800

[36] Perišić Nanut M Sabotič J Jewett A Kos J. Cysteine cathepsins as regulators of the cytotoxicity of NK and T cells. Front Immunol (2014) 5:616. doi: 10.3389/fimmu.2014.00616 25520721 PMC4251435

[37] Wen T Barham W Li Y Zhang H Gicobi JK Hirdler JB. NKG7 is a T-cell-Intrinsic therapeutic target for improving antitumor cytotoxicity and cancer immunotherapy. Cancer Immunol Res (2022) 10:162–81. doi: 10.1158/2326-6066.CIR-21-0539 PMC8816890 34911739

[38] Yang B-H Wang K Wan S Liang Y Yuan X Dong Y. TCF1 and LEF1 control treg competitive survival and tfr development to prevent autoimmune diseases. Cell Rep (2019) 27:3629–3645.e6. doi: 10.1016/j.celrep.2019.05.061 31216480 PMC6701704

[39] Mo X-D Xu L-P Zhang X-H Liu D-H Wang Y Chen H. Chronic GVHD induced GVL effect after unmanipulated haploidentical hematopoietic SCT for AML and myelodysplastic syndrome. Bone Marrow Transplant (2015) 50:127–33. doi: 10.1038/bmt.2014.223 25387095

[40] Weisdorf D Zhang M-J Arora M Horowitz MM Rizzo JD Eapen M. Graft-versus-host disease induced graft-versus-leukemia effect: Greater impact on relapse and disease-free survival after reduced intensity conditioning. Biol Blood Marrow Transplant J Am Soc Blood Marrow Transplant (2012) 18:1727–33. doi: 10.1016/j.bbmt.2012.06.014 PMC3472079 22766220

[41] Song Y Hu B Liu Y Jin Z Zhang Y Lin D. IL-12/IL-18-preactivated donor NK cells enhance GVL effects and mitigate GvHD after allogeneic hematopoietic stem cell transplantation. Eur J Immunol (2018) 48:670–82. doi: 10.1002/eji.201747177 29282719

[42] Jiang H Fu D Bidgoli A Paczesny S. T Cell subsets in graft versus host disease and graft versus tumor. Front Immunol (2021) 12:761448. doi: 10.3389/fimmu.2021.761448 34675938 PMC8525316

[43] Tremblay-McLean A Coenraads S Kiani Z Dupuy FP Bernard NF. Expression of ligands for activating natural killer cell receptors on cell lines commonly used to assess natural killer cell function. BMC Immunol (2019) 20:8. doi: 10.1186/s12865-018-0272-x 30696399 PMC6352444

[44] Bigley AB Rezvani K Chew C Sekine T Pistillo M Crucian B. Acute exercise preferentially redeploys NK-cells with a highly-differentiated phenotype and augments cytotoxicity against lymphoma and multiple myeloma target cells. Brain Behav Immun (2014) 39:160–71. doi: 10.1016/j.bbi.2013.10.030 24200514

[45] Matsuda M Ono R Iyoda T Endo T Iwasaki M Tomizawa-Murasawa M. Human NK cell development in hIL-7 and hIL-15 knockin NOD/SCID/IL2rgKO mice. Life Sci Alliance (2019) 2:e201800195. doi: 10.26508/lsa.201800195 30936185 PMC6445396

[46] Kurz E Hirsch CA Dalton T Shadaloey SA Khodadadi-Jamayran A Miller G. Exercise-induced engagement of the IL-15/IL-15Rα axis promotes anti-tumor immunity in pancreatic cancer. Cancer Cell (2022) 40(7):S1535–6108(22)00217-3. doi: 10.1016/j.ccell.2022.05.006 PMC9280705 35660135

[47] Chen Y Chen B Yang T Xiao W Qian L Ding Y. Human fused NKG2D-IL-15 protein controls xenografted human gastric cancer through the recruitment and activation of NK cells. Cell Mol Immunol (2017) 14:293–307. doi: 10.1038/cmi.2015.81 26364916 PMC5360879

[48] LaVoy ECP Bollard CM Hanley PJ Blaney JW O’Connor DP Bosch JA. A single bout of dynamic exercise enhances the expansion of MAGE-A4 and PRAME-specific cytotoxic T-cells from healthy adults. Exerc Immunol Rev (2015) 21:144–53. 25826370

[49] Baker FL Bigley AB Agha NH Pedlar CR O’Connor DP Bond RA. Systemic β-adrenergic receptor activation augments the *ex vivo* expansion and anti-tumor activity of Vγ9Vδ2 T-cells. Front Immunol (2019) 10:3082. doi: 10.3389/fimmu.2019.03082 32038628 PMC6993603

