# Supplementary figures and images for: Human lymphocytes mobilized with exercise have an anti-tumor transcriptomic profile and exert enhanced graft-versus-leukemia effects in xenogeneic mice
Source: Front Immunol. 2023 Apr 3;14:1067369. doi: 10.3389/fimmu.2023.1067369 (PMC10109447; doi:10.3389/fimmu.2023.1067369)

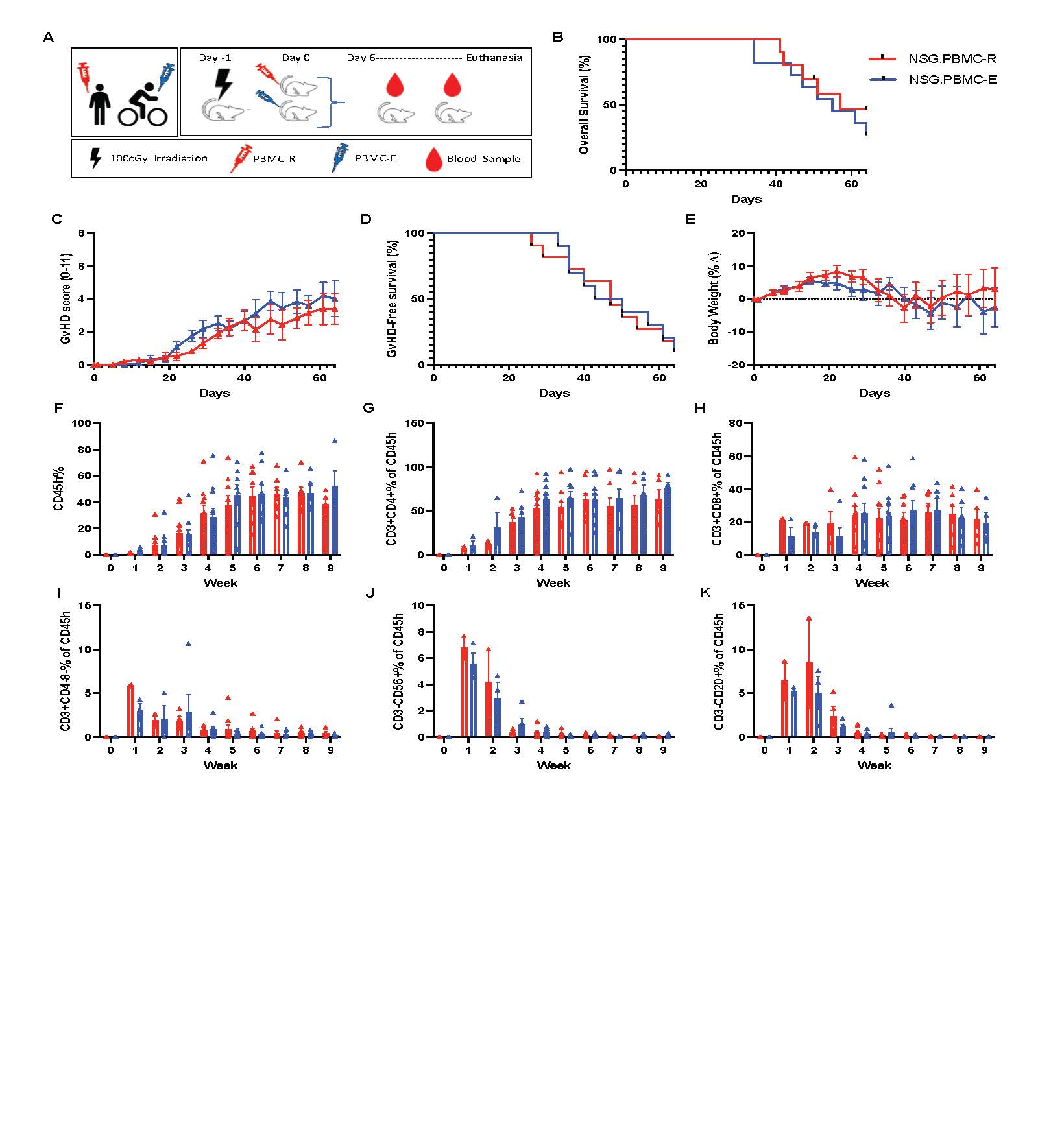

Supplement: Supplementary Figure 1 — Exercise-mobilized PBMCs have no effect on survival, GvHD or human immune cell engraftment in non-tumor bearing standard NSG mice. To determine the GvHD effect of the PBMCs mobilized by exercise in a less severe model, standard NSG mice were injected with PBMCs (10x106) collected either at rest (PBMC-R) or during (PBMC-E) exercise. (A) Illustration of the experimental design. (B) Overall survival. (C) GvHD score measured twice weekly. (D) GvHD-free survival determined using a composite score of ≥4. (E) body weight. To track the human immune cell engraftment, mouse blood was collected weekly starting at Day+7 and whole blood was labeled with monoclonal antibodies to enumerate the following human cell types: (F) CD45h, (G) CD4+ T cells, (H) CD8+ T cells, (I) DN T cells (J) NK cells, and (K) B cells. There were no significant differences between PBMC-R and PBMC-E (p>0.05). (n=11 mice/group). [file Image_1.jpeg]
