# Supplementary material for: Human lymphocytes mobilized with exercise have an anti-tumor transcriptomic profile and exert enhanced graft-versus-leukemia effects in xenogeneic mice
Source: Front Immunol. 2023 Apr 3;14:1067369. doi: 10.3389/fimmu.2023.1067369 (PMC10109447; doi:10.3389/fimmu.2023.1067369)
Supplement: Supplementary file 2 [file Table_1.docx]

Supplementary table 1 – Phenotype panels of cell surface markers.

|  | **V1**  **VioBlue** | **V2**  **VioGreen** | **B1**  **FITC** | **B2**  **PE** | **B3**  **PerCP** | **B4**  **PE-Vio770** | **R1**  **APC** | **R2**  **APC-Vio 770** |
| --- | --- | --- | --- | --- | --- | --- | --- | --- |
| **Panel 1** | CD8 | CD14 | CD3 | CD4 | CD20 |  | CD45 | CD56 |
| **Panel 2** | CD8 | CD3 | CD4 | CD62L | PD-1 | CD45Ra | CD45 | CD56 |
| **Panel 3** | CD8 | CD3 | TCR-Vδ2 | CD4 | CD45 | TCR-αβ | TCR-Vδ1 | TCR-γδ |
| **Panel 4** | CD57 | CD3 | NKG2C | NKG2D | CD45 | NKp46 | NKG2A | CD56 |
| **Panel 5** | CD16 | CD3 | CD69 | NKp30 | CD158a | CD158b | CD45 | CD56 |
| **Panel 6** | CD8 | CD3 | CD4 | CD27 | PD-1 | CD28 | iNKT | CD45 |
| **Panel 7** | CD8 | CD3 | CD127 | CD25 | PI |  | CD4 | CD45 |
| **Panel 8** | CD16 | CD14 | CD15 | CD33 | CD11b | HLA.DR | CD11c | CD45 |
