# Supplementary material for: Human lymphocytes mobilized with exercise have an anti-tumor transcriptomic profile and exert enhanced graft-versus-leukemia effects in xenogeneic mice
Source: Front Immunol. 2023 Apr 3;14:1067369. doi: 10.3389/fimmu.2023.1067369 (PMC10109447; doi:10.3389/fimmu.2023.1067369)
Supplement: Supplementary file 3 [file Table_2.docx]

Supplementary table 2 - Differentially expressed genes in lymphocytes and monocytes in response to exercise as determined by single cell RNAseq. GeneCards®: the human gene database was utilized to identify gene name and function (Stelzer, 2016). Upwards arrows (↑) indicate differentially expressed gene was upregulated between timepoints; downwards arrow (↓) indicates differentially expressed gene was downregulated between timepoints; double-sided arrows (↔) indicate there was no significant change in gene expression between timepoints.

| Gene | Name | Function | CD8 | CD8 EM | CD4 | CD4 CM | NK | MAIT | Mono |
| --- | --- | --- | --- | --- | --- | --- | --- | --- | --- |
| ADGRE5 | Adhesion G Protein-Coupled Receptor E5 | Plays a role in cell adhesion and leukocyte recruitment, activation, and migration. | ↑ | ↔ | ↔ | ↔ | ↔ | ↔ | ↔ |
| ANXA1 | Annexin A1 | Membrane-localized protein that binds phospholipids. This protein inhibits phospholipase A2 and has anti-inflammatory activity. | ↑ | ↑ | ↑ | ↑ | ↔ | ↔ | ↔ |
| APOBEC3G | Apolipoprotein B MRNA Editing Enzyme Catalytic Subunit 3G | Member of the cytidine deaminase gene family that catalyzes site-specific deamination of both RNA and single-stranded DNA. | ↑ | ↑ | ↔ | ↔ | ↔ | ↔ | ↔ |
| ARL4C | ADP Ribosylation Factor Like GTPase 4C | Encodes a protein member of the ADP-ribosylation factor family of GTP-binding proteins. This protein may play a role in cholesterol transport. | ↑ | ↔ | ↔ | ↔ | ↔ | ↔ | ↔ |
| B2M | Beta-2-Microglobulin | Encodes a serum protein found in association with MHC class I heavy chain. It displays antibacterial activity in amniotic fluid. | ↔ | ↔ | ↔ | ↔ | ↓ | ↔ | ↔ |
| BACH2 | BTB Domain and CNC Homolog 2 | Protein Coding gene. Among its related pathways are NF-kappaB Signaling. | ↓ | ↔ | ↔ | ↔ | ↔ | ↔ | ↔ |
| CALM1 | Calmodulin 1 | This gene encodes one of three calmodulin proteins that regulates and modulates the function of cardiac ion channels. | ↑ | ↔ | ↔ | ↔ | ↔ | ↔ | ↔ |
| CCL5 | C-C Motif Chemokine Ligand 5 | This chemokine, a member of the CC subfamily, functions as a chemoattractant for blood monocytes, memory T helper cells and eosinophils. | ↑ | ↔ | ↔ | ↔ | ↔ | ↔ | ↔ |
| CCR7 | C-C Motif Chemokine Receptor 7 | This receptor is expressed in various lymphoid tissues and activates B and T lymphocytes. It controls the migration of memory T cells to inflamed tissues and stimulate dendritic cell maturation. | ↓ | ↔ | ↔ | ↔ | ↔ | ↔ | ↔ |
| CD300A | CD300a Molecule | Member of the CD300 glycoprotein family of cell surface proteins found on leukocytes. It is involved in immune response signaling pathways. | ↑ | ↔ | ↔ | ↔ | ↔ | ↔ | ↔ |
| CD3E | CD3e Molecule | Plays a role in coupling antigen recognition to several intracellular signal-transduction pathways. | ↔ | ↔ | ↔ | ↔ | ↓ | ↔ | ↔ |
| CD55 | CD55 Molecule | Encodes a glycoprotein involved in the regulation of the complement cascade. | ↓ | ↔ | ↔ | ↔ | ↔ | ↔ | ↔ |
| CD58 | CD58 Molecule | Member of the immunoglobulin superfamily. Ligand of the T lymphocyte CD2 protein, and functions in adhesion and activation of T lymphocytes. ͍ | ↑ | ↔ | ↔ | ↔ | ↔ | ↔ | ↔ |
| CD63 | CD63 Molecule | Cell surface glycoprotein that is known to complex with integrins. It may function as a blood platelet activation marker. | ↑ | ↔ | ↔ | ↔ | ↔ | ↔ | ↔ |
| CD99 | CD99 Molecule (Xg Blood Group) | Cell surface glycoprotein involved in leukocyte migration, T-cell adhesion, ganglioside GM1 and transmembrane protein transport, and T-cell death by a caspase-independent pathway. | ↑ | ↔ | ↔ | ↔ | ↔ | ↔ | ↔ |
| CST7 | Cystatin F | Plays a role in immune regulation through inhibition of a unique target in the hematopoietic system. | ↑ | ↑ | ↔ | ↔ | ↔ | ↔ | ↔ |
| CX3CR1 | C-X3-C Motif Chemokine Receptor 1 | Encodes a receptor for fractalkine. Fractalkine is a transmembrane protein and chemokine involved in the adhesion and migration of leukocytes. | ↑ | ↔ | ↔ | ↔ | ↔ | ↔ | ↔ |
| CXCR4 | C-X-C Motif Chemokine Receptor 4 | Encodes a CXC chemokine receptor specific for stromal cell-derived factor-1. | ↔ | ↔ | ↔ | ↔ | ↓ | ↔ | ↔ |
| DUSP2 | Dual Specificity Phosphatase 2 | Encodes a member of a phosphatase subfamily, that negatively regulate members of the MAP-kinase superfamily, which are associated with cellular proliferation and differentiation. | ↑ | ↔ | ↔ | ↔ | ↔ | ↔ | ↔ |
| EGR1 | Early Growth Response 1 | Nuclear protein. It functions as a transcriptional regulator | ↔ | ↔ | ↔ | ↔ | ↔ | ↔ | ↑ |
| FCGR3A | Fc Gamma Receptor IIIa | Encodes a receptor for the Fc portion of immunoglobulin G, and it is involved in the removal of antigen-antibody complexes from the circulation. | ↑ | ↑ | ↔ | ↔ | ↔ | ↔ | ↔ |
| FOXP1 | Forkhead Box P1 | Plays important roles in the regulation of tissue- and cell type-specific gene transcription during both development and adulthood. | ↓ | ↔ | ↔ | ↔ | ↔ | ↔ | ↔ |
| GNG2 | G Protein Subunit Gamma 2 | Encodes one of the gamma subunits of a guanine nucleotide-binding protein involved in signaling mechanisms across membranes. | ↔ | ↔ | ↔ | ↔ | ↑ | ↔ | ↔ |
| GNLY | Granulysin | Antimicrobial activity. It is present in cytotoxic granules of cytotoxic T lymphocytes and natural killer cells. | ↑ | ↑ | ↔ | ↔ | ↓ | ↔ | ↔ |
| GZMA | Granzyme A | May function as a common component necessary for lysis of target cells by cytotoxic T lymphocytes and natural killer cells. | ↑ | ↑ | ↔ | ↔ | ↔ | ↔ | ↔ |
| GZMB | Granzyme B | Secreted by natural killer (NK) cells and cytotoxic T lymphocytes (CTLs) to induce target cell apoptosis. | ↑ | ↑ | ↔ | ↔ | ↑ | ↔ | ↔ |
| GZMH | Granzyme H | Constitutively expressed in natural killer cells, induces target cell death by directly cleaving substrates in pathogen-infected cells | ↑ | ↑ | ↔ | ↔ | ↔ | ↔ | ↔ |
| GZMM | Granzyme M | Subset of neutral serine proteases that is expressed and stored by Human NK cells and activated lymphocytes. | ↑ | ↔ | ↔ | ↔ | ↔ | ↔ | ↔ |
| HLA-C | Major Histocompatibility Complex, Class I, C | HLA-C belongs to the HLA class I heavy chain paralogues and present peptides derived from endoplasmic reticulum lumen. | ↔ | ↔ | ↔ | ↔ | ↓ | ↔ | ↔ |
| HLA-DPA1 | Major Histocompatibility Complex, Class II, DP Alpha 1 | This gene belongs to the HLA class II alpha chain paralogues. It plays a central role in the immune system by presenting peptides derived from extracellular proteins. | ↑ | ↔ | ↔ | ↔ | ↔ | ↔ | ↔ |
| IFITM1 | Interferon Induced Transmembrane Protein 1 | Family of interferon induced antiviral proteins. Restricts cellular entry by diverse viral pathogens | ↔ | ↔ | ↔ | ↔ | ↔ | ↓ | ↔ |
| IFITM2 | Interferon Induced Transmembrane Protein 2 | Restricts cellular entry by diverse viral pathogens, such as influenza A virus, Ebola virus and Sars-CoV-2. | ↑ | ↔ | ↔ | ↔ | ↔ | ↓ | ↔ |
| IL32 | Interleukin 32 | Expression of this protein is increased after the activation of T-cells by mitogens or the activation of NK cells by IL-2. This protein induces the production of TNFalpha from macrophage cells. | ↑ | ↔ | ↔ | ↔ | ↔ | ↔ | ↔ |
| IL7R | Interleukin 7 Receptor | Encodes a receptor for interleukin 7 (IL7). Plays a critical role in V(D)J recombination during lymphocyte development. | ↓ | ↔ | ↔ | ↔ | ↔ | ↔ | ↔ |
| IRF1 | Interferon Regulatory Factor 1 | Plays a role in cell proliferation, apoptosis, the immune response, and DNA damage response. This protein represses the transcription of several other genes. | ↑ | ↔ | ↔ | ↔ | ↔ | ↔ | ↔ |
| ITGB1 | Integrin Subunit Beta 1 | Encodes a beta subunit of Integrins. Integrin family members are membrane receptors involved in cell adhesion and recognition in a variety of processes including embryogenesis, hemostasis, tissue repair, immune response and metastatic diffusion of tumor cells. | ↑ | ↔ | ↔ | ↔ | ↔ | ↔ | ↔ |
| ITGB2 | Integrin Subunit Beta 2 | Encodes an integrin beta chain. It plays an important role in immune response. Defects in this gene cause leukocyte adhesion deficiency. | ↑ | ↑ | ↔ | ↔ | ↔ | ↔ | ↔ |
| JUN | Jun Proto-Oncogene, AP-1 Transcription Factor Subunit | Interacts directly with specific target DNA sequences to regulate gene expression. | ↔ | ↔ | ↑ | ↑ | ↔ | ↔ | ↔ |
| KIR2DL3 | Killer Cell Immunoglobulin Like Receptor, Two Ig Domains and Long Cytoplasmic Tail 3 | Transmembrane glycoproteins expressed by natural killer cells and subsets of T cells that plays an important role in regulation of the immune response. | ↑ | ↔ | ↔ | ↔ | ↔ | ↔ | ↔ |
| KLRD1 | Killer Cell Lectin Like Receptor D1 | Antigen preferentially expressed on NK cells. | ↑ | ↔ | ↔ | ↔ | ↔ | ↔ | ↔ |
| LAG3 | Lymphocyte Activating 3 | Belongs to Ig superfamily and contains 4 extracellular Ig-like domains. | ↑ | ↔ | ↔ | ↔ | ↔ | ↔ | ↔ |
| LEF1 | Lymphoid Enhancer-Binding Factor 1 | Binds to a functionally important site in the T-cell receptor-alpha enhancer, conferring maximal enhancer activity | ↓ | ↓ | ↔ | ↔ | ↔ | ↔ | ↔ |
| LGALS1 | Galectin 1 | Proteins implicated in modulating cell-cell and cell-matrix interactions. May act as an autocrine negative growth factor that regulates cell proliferation. | ↑ | ↑ | ↔ | ↔ | ↔ | ↔ | ↔ |
| LTB | Lymphotoxin Beta | Type II membrane protein of the TNF family. Inducer of the inflammatory response system and involved in normal development of lymphoid tissue. | ↓ | ↓ | ↔ | ↔ | ↔ | ↔ | ↔ |
| MALAT1 | Metastasis Associated Lung Adenocarcinoma Transcript 1 | Involved in cell cycle regulation. May act as a transcriptional regulator of genes involved in cancer metastasis and cell migration. | ↓ | ↓ | ↓ | ↓ | ↔ | ↔ | ↔ |
| NKG7 | Natural Killer Cell Granule Protein 7 | [Protein Coding gene. Diseases associated with NKG7 include Leukemia, Chronic Myeloid.](https://www.malacards.org/card/leukemia_chronic_myeloid) | ↑ | ↑ | ↔ | ↔ | ↓ | ↔ | ↔ |
| NPM1 | Nucleophosmin 1 | Involved in several cellular processes, including centrosome duplication, protein chaperoning, and cell proliferation. | ↓ | ↓ | ↔ | ↔ | ↔ | ↔ | ↔ |
| PDE4D | Phosphodiesterase 4D | The encoded protein has 3',5'-cyclic-AMP phosphodiesterase activity and degrades cAMP, which acts as a signal transduction molecule in multiple cell types. | ↑ | ↔ | ↔ | ↔ | ↔ | ↔ | ↔ |
| PIK3IP1 | Phosphoinositide-3-Kinase-Interacting Protein 1 | Protein Coding gene | ↓ | ↓ | ↔ | ↔ | ↔ | ↔ | ↔ |
| PIK3R1 | Phosphoinositide-3-Kinase Regulatory Subunit 1 | hosphatidylinositol 3-kinase plays an important role in the metabolic actions of insulin, and a mutation in this gene has been associated with insulin resistance. | ↑ | ↔ | ↔ | ↔ | ↔ | ↔ | ↔ |
| PRF1 | Perforin 1 | This protein forms membrane pores that allow the release of granzymes and subsequent cytolysis of target cells. | ↑ | ↑ | ↔ | ↔ | ↔ | ↔ | ↔ |
| RPL28 | Ribosomal Protein L28 | This gene encodes a ribosomal protein that is a component of the 60S subunit. | ↓ | ↔ | ↔ | ↔ | ↔ | ↔ | ↔ |
| RPL9 | Ribosomal Protein L9 | This gene encodes a ribosomal protein that is a component of the 60S subunit. | ↓ | ↔ | ↔ | ↔ | ↔ | ↔ | ↔ |
| RPLP1 | Ribosomal Protein Lateral Stalk Subunit P1 | This gene encodes a ribosomal phosphoprotein that is a component of the 60S subunit. | ↔ | ↔ | ↔ | ↔ | ↔ | ↔ | ↓ |
| RPS12 | Ribosomal Protein S12 | This gene encodes a ribosomal protein that is a component of the 40S subunit. | ↓ | ↔ | ↔ | ↔ | ↔ | ↔ | ↔ |
| RPS23 | Ribosomal Protein S23 | This gene encodes a ribosomal protein that is a component of the 40S subunit. | ↓ | ↓ | ↔ | ↔ | ↔ | ↔ | ↔ |
| RUNX3 | RUNX Family Transcription Factor 3 | Member of the runt domain-containing family of transcription factors. It functions as a tumor suppressor, and the gene is frequently deleted or transcriptionally silenced in cancer. | ↑ | ↔ | ↔ | ↔ | ↔ | ↔ | ↔ |
| S100A10 | S100 Calcium Binding Protein A10 | Encodes a protein member of the S100 family. it is involved in the regulation of several cellular processes such as cell cycle progression and differentiation. | ↑ | ↔ | ↔ | ↔ | ↔ | ↔ | ↔ |
| SELL | Selectin L | Cell surface adhesion molecule. Required for binding and rolling of leucocytes on endothelial cells, facilitating their migration into secondary lymphoid organs and inflammation sites. | ↓ | ↔ | ↔ | ↔ | ↔ | ↔ | ↔ |
| TBX21 | T-Box Transcription Factor 21 | This gene is the human ortholog of mouse Tbx21/Tbet gene. Tbx21 protein is a Th1 cell-specific transcription factor that controls interferon-gamma expression. | ↑ | ↔ | ↔ | ↔ | ↔ | ↔ | ↔ |
| TCF7 | Transcription Factor 7 | This gene is expressed predominantly in T-cells and plays a critical role in natural killer cell and innate lymphoid cell development. | ↓ | ↔ | ↔ | ↔ | ↔ | ↔ | ↔ |
| TGFB1 | Transforming Growth Factor Beta 1 | Encodes a secreted ligand of the TGF-beta superfamily of proteins. It regulates cell proliferation, differentiation and growth, and can modulate expression and activation of other growth factors including interferon gamma and tumor necrosis factor alpha. | ↑ | ↔ | ↔ | ↔ | ↔ | ↔ | ↔ |
| TIGIT | T Cell Immunoreceptor with Ig And ITIM Domains | Encodes a member of the PVR (poliovirus receptor) family of immunoglobin proteins that is expressed on several classes of T cells including follicular B helper T cells (TFH). | ↑ | ↑ | ↔ | ↔ | ↔ | ↔ | ↔ |
| TXNIP | Thioredoxin Interacting Protein | Inhibits the antioxidative function of thioredoxin resulting in the accumulation of reactive oxygen species and cellular stress | ↓ | ↓ | ↓ | ↓ | ↔ | ↔ | ↔ |
