# Supplementary material for: Human lymphocytes mobilized with exercise have an anti-tumor transcriptomic profile and exert enhanced graft-versus-leukemia effects in xenogeneic mice
Source: Front Immunol. 2023 Apr 3;14:1067369. doi: 10.3389/fimmu.2023.1067369 (PMC10109447; doi:10.3389/fimmu.2023.1067369)
Supplement: Supplementary file 4 [file Table_3.docx]

Supplementary table 3 – NES, P value and FDR value for the KEGG terms associated with enhanced anti-tumor activity in CD8 T cells and NK cells.

| **KEGG Term** | **CD8** | | | **NK** | | |
| --- | --- | --- | --- | --- | --- | --- |
|  | **NES** | **NOM p-val** | **FDR q-val** | **NES** | **NOM p-val** | **FDR q-val** |
| NATURAL KILLER CELL MEDIATED CYTOTOXICITY%KEGG%HSA04650 | 1.893552 | 0 | 0 | 1.558397 | 0 | 0.356428 |
| GRAFT-VERSUS-HOST DISEASE%KEGG%HSA05332 | 1.87739 | 0 | 0 | 1.193661 | 0.169014 | 0.236071 |
| ALLOGRAFT REJECTION%KEGG%HSA05330 | 1.827106 | 0 | 0 | 1.245683 | 0.107955 | 0.958057 |
| ANTIGEN PROCESSING AND PRESENTATION%KEGG%HSA04612 | 1.713356 | 0 | 0.013984 | - | - | - |
| CELL ADHESION MOLECULES (CAMS)%KEGG%HSA04514 | 1.653069 | 0.002 | 0.036855 | - | - | - |
